# 923. Respiratory Syncytial and Parainfluenza Virus Infection Increase the Risk of Cytomegalovirus Reactivation in Allogeneic Hematopoietic Cell Transplant Recipients

**DOI:** 10.1093/ofid/ofab466.1118

**Published:** 2021-12-04

**Authors:** Hu Xie, Alpana Waghmare, Guang-Shing Cheng, Leonidas Stamatatos, Keith Jerome, Wendy Leisenring, Janet A Englund, Michael Boeckh, Chikara Ogimi

**Affiliations:** 1 Fred Hutchinson Cancer Research Center; University of Washington, Seattle, Washington; 2 Seattle Children’s Hospital/University of Washington/Fred Hutchinson Cancer Research Center, Seattle, Washington; 3 Fred Hutchinson Cancer Research Center, Seattle, Washington; 4 University of Washington, Seattle, WA; 5 Seattle Children’s Hospital/Univ. of Washington, Seattle, Washington

## Abstract

**Background:**

Respiratory virus infections are associated with significant and specific local and systemic inflammatory response patterns, which may lead to reactivation of latent viruses. We examined whether viral upper (URTI) or lower respiratory tract infection (LRTI) with common respiratory viruses increased the risk of CMV viremia after allogeneic hematopoietic cell transplantation (HCT).

**Methods:**

We retrospectively analyzed patients undergoing allogeneic HCT between 4/2008 and 9/2018. CMV surveillance was performed weekly and the presence of upper and lower respiratory symptoms were evaluated by multiplex respiratory viral PCR. We used Cox proportional hazards models to evaluate risk factors for development of any CMV viremia or high level CMV viremia in the first 100 days post-HCT. Each respiratory virus infection episode was considered positive for 30 days beginning the day of diagnosis.

**Results:**

Among 2,545 patients (404 children, 2141 adults), 1,221 and 247 developed CMV viremia and high level CMV viremia, respectively, in the first 100 days post-HCT. Infections due to human rhinoviruses (HRV, N=476) were most frequent, followed by parainfluenza viruses 1-4 (PIV, N=139), seasonal human coronaviruses (COV, N=134), respiratory syncytial virus (RSV, N=77), influenza A/B (FLU, N=35), human metapneumovirus (MPV, N=37), and adenovirus (ADV, N=61). In adjusted models, RSV LRTI was associated with increased risk of developing CMV viremia at all levels (**Figures 1** and **2**), and PIV or RSV URTI increased the risk of high level CMV viremia; all other viruses showed no association in univariable models.

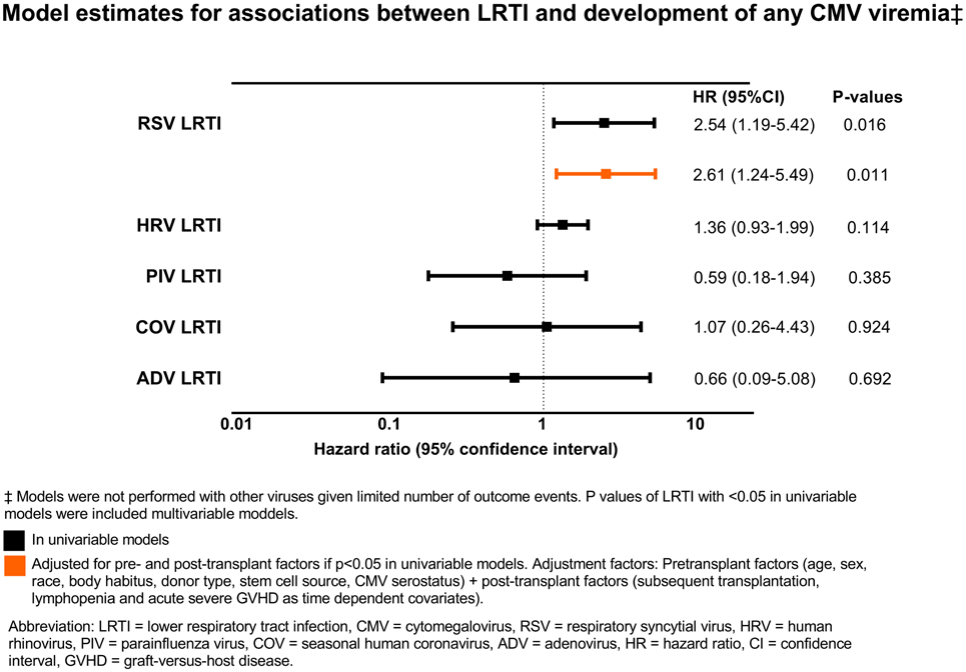

Figure 1. Model estimates for associations between LRTI and development of any CMV viremia

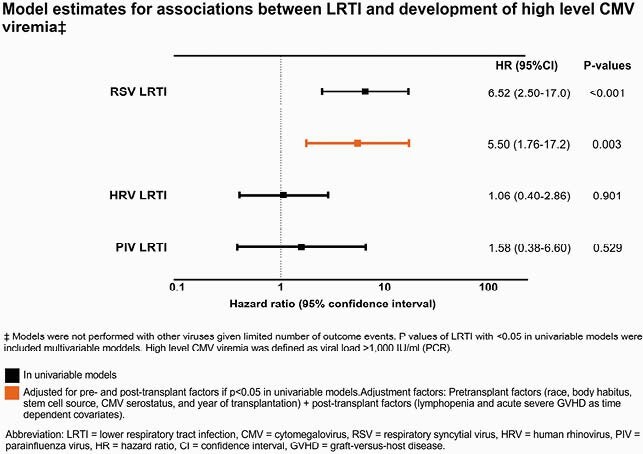

Figure 2. Model estimates for associations between LRTI and development of high level CMV viremia

**Conclusion:**

We demonstrated that RSV and PIV infections are associated with an increased risk for development of CMV viremia after allogeneic HCT. This novel association provides the rationale to explore virus-specific inflammatory pathways that may trigger CMV reactivation. CMV viremia may also serve as an endpoint in clinical trials that assess new preventative or therapeutic interventions of RSV or PIV infection.

**Disclosures:**

**Alpana Waghmare, MD**, **Allovir** (Scientific Research Study Investigator)**Ansun Biopharma** (Scientific Research Study Investigator)**Kyorin Pharmaceutical** (Advisor or Review Panel member) **Janet A. Englund, MD**, **AstraZeneca** (Consultant, Grant/Research Support)**GlaxoSmithKline** (Research Grant or Support)**Meissa Vaccines** (Consultant)**Pfizer** (Research Grant or Support)**Sanofi Pasteur** (Consultant)**Teva Pharmaceuticals** (Consultant) **Michael Boeckh, MD PhD**, **AlloVir** (Consultant)**Ansun Biopharma** (Grant/Research Support)**Astellas** (Grant/Research Support)**EvrysBio** (Consultant, Other Financial or Material Support, Options to acquire equity, but have not exercised them)**Gilead Sciences** (Consultant, Grant/Research Support)**GlaxoSmithKline** (Consultant)**Helocyte** (Consultant, Other Financial or Material Support, Options to acquire equity, but have not exercised them)**Janssen** (Grant/Research Support)**Kyorin** (Consultant)**Merck** (Consultant, Grant/Research Support)**Moderna** (Consultant)**Symbio** (Consultant)**Takeda (formerly known as Shire**) (Consultant, Grant/Research Support)**VirBio** (Consultant, Grant/Research Support)

